# A Series of Tracheotomies

**Published:** 1906-06

**Authors:** George F. Cott

**Affiliations:** Buffalo, N. Y.; Clinical Professor of Otology, University of Buffalo. 1248 Main Street


### A Series of Tracheotomies.

By GEORGE F. COTT, M. D., Buffalo, N. Y.
Clinical Professor of Otology, University of Buffalo.

TRACHEOTOMY is not nearly so important to the surgeon as to the patient, yet there are certain circumstances connected with the operation, or rather conditions existing immediately before, which may well bear being told over and over again, especially to those who do not meet many such cases. I believe I have been especially favored in being called upon to operate upon patients who were choking from one cause or another. I don't think it is usually the lot of laryngologists to be called upon very often to officiate in cases where tracheotomy is required, but rather the general surgeon. Then again so many cases are treated at hospitals where everything is much more convenient that private practice is not at all crowded with this particular kind of cases. Tracheotomy is so seldom called for that the physician is not always prepared for the emergency.

I doubt if, on an average, one case occurs to every physician during his life-time. He who is so unfortunate as to meet with such a case wends his way to some one who is supposed to be ready for any emergency. I have had the good fortune to be called to several cases "in extremis" and was able to tide them over the danger point even though some were apparently beyond the aid of the surgeon, while a few succumbed after the throat had been opened. As I considered cases of choking, where tracheotomy would be indicated, were rather rare, I did not provide myself with the necessary instruments until once I was caught napping and had to operate with a knife only; this was rather cumbersome, and after that I carried instruments and tubes with my regular outfit for intubation. I never had reason to regret that precaution for several times the windpipe was just opened in the nick of time to save the patient's life.

When we open a windpipe it is always for the purpose of relieving some obstruction in the larynx or immediately below it, unless some foreign body was accidently drawn down into the trachea or the latter pressed upon by some growth. In all cases stenosis was due to disease of some kind affecting the larynx or subglottic tissue except one which will presently be mentioned. I may add that tracheotomy is not always easily performed especially in a little child with a fat neck, as we often find in acute cases.

The efforts at inspiration draw the trachea away from its normal position so that it is difficult to locate, and sometimes careful dissection is necessary to find and hold it long enough to open and introduce a tube. Then again the light is often bad, the room close, (for you must remember these are generally emergency cases), assistants are often wanting; father, mother, some relative or neighbor usually assist, in a way, of course. The physician in charge administers chloroform, and must give his entire attention to the anesthetic. The operator must depend mostly upon his own ingenuity in bringing the work to a successful issue. It also happens that one of the inexperienced assistants takes it into his head to go off in a dead faint, and drops the lamp with a smash, as happened in one of my operations. All these little by-plays are very annoying and handicap the surgeon greatly. But his duty is to save the life of the patient, regardless of such unforseen incidental happenings. The time in which over a hundred patients were intubated, tracheotomy became necessary in twenty-six, either with or without intubation; a résumé of these cases follows:

CASE 1: A little boy, age 2 1-2. Intubated, but at end of twenty-four hours tube was extracted as child labored for breath. During removal, child stopped breathing, artificial respiration became necessary until breathing was re-established. Tracheotomy was then hurridly done, with no other instrument but a scalpel. After the trachea was opened no more air was admitted than before and the child died on the table. This was the first and only time I was caught unprepared.

CASE 2: A little boy, age 2 1-2, in the practice of Dr. Thompson. Intubated by Dr. Potter several times for diphtheritic stenosis. At last a 7 year tube only could be retained. Dr. Potter being out of town, I was called to re-intubate, the child having coughed up the tube, and then recommended tracheotomy which was done. This little child, who is now about eight years old, was unfortunate enough to have granulation tissue form within the trachea, so that he had to wear either a tracheal or O'Dwyer tube ever since. At age of 10 accidently coughed up tube and choked before assistance could be given.

CASE 3: Mr. R. age 35. Tubercular subglottic laryngitis causing stenosis. Let me remind you, gentlemen, that tubercular laryngitis rarely calls for intubation or tracheotomy, yet in my short career I was called upon to operate three times for this disease. In this case, trachea was opened under chloroform. Patient did well for several months; lungs and sputum examined several times without result. One day during a fit of coughing he lay back in bed and suddenly died. Post-mortem revealed miliary tuberculosis and both bronchi accluded by a plug of tenaceous mucopus.

CASE 4: Man, age 31, practice of Dr. Gibson. Stenosis somewhere in the trachea. It could not be made out with the mirror. Patient was to walk from one room to another, where a table was prepared, but suddenly dropped to the floor and stopped breathing. He was hurriedly carried to the table, and a knife plunged into his throat, cutting down to the trachea, which was opened and a tube inserted. The patient was then held, head downward, to let the blood escape from the windpipe, when he began to breathe again. This gentleman weighed 180 pounds, and was in the best of health until about six hours before the operation. The cause of stenosis was erythema nodosum affecting the tracheal mucous membrane. Diagnosis made by Dr. Grover Wende after the operation. Patient recovered in a week.

CASE 5: Man age 35. Tubercular laryngitis involving the cords but principally subglottic. Sent him to the Fitch Hospital to be watched over night, but found it necessary to intubate. Coughed up tube three times, and began choking severely. Put him on the table to administer chloroform, but as he stopped breathing I had to do a rapid tracheotomy. It was done with one sweep of the knife, the tissue being so soft that I could work readily with my fingers, open the trachea and insert a tube when the patient began to breathe again. He died some weeks later in the country of pulmonary tuberculosis.

CASE. 6: Boy age 2 1-2. Diphtheria. In the practice of Dr. Ray H. Johnson. Intubated, but the tube was coughed up during my absence. When I arrived the patient was about gone, and a hurried tracheotomy was performed, when he again breathed. With much care on the part of Dr. Johnson, the child made a tedious but final recovery. Dr. Johnson took every precaution to prevent carrying infection to his patients or becoming infected himself, by wearing a hospital gown and using strong disinfectants. However shortly after, he took the disease from the effects of which he suffered for three months, and was unable to attend his patients during that time.

CASE 7: Man age 60. Carcinoma of larynx. Tracheotomy under cocaine. Tube was taken out to be cleaned by an assistant next day. After working for an hour, he found he could not reintroduce it. Upon examination I found what the cause was; the trachea had been drawn to one side while being opened and therefore when the tube was taken out, the opening became hidden under the tissue. Thus the difficulty.

CASE 8: Mrs. B, age 34. Acute subglottic edema, due to exposure to cold. After suffering several days, called at Dr. Dorr's office, who recognising her serious condition, and telephoned me to see her. Laryngoscopic examination caused a severe spasm and I proposed immediate tracheotomy, which was done under cocaine anesthesia. Several times while wearing the tube it became occluded with inspissated mucous and threatened suffocation. Gradual recovery.

CASE 9: A little Italian baby, about a year old. Diagnosis: acute laryngitis with subglottic edema. Tried to intubate at College dispensary, but the tube would not go down. After several attempts the child was threatened with suffocation. I hurried it over to the Buffalo General Hospital and just had time enough to open the trachea to prevent death. Removed the tube after eight days, but could not intubate, there still being obstruction above the tracheal wound. After another week the tube was again removed, the larynx was found clear, and the child recovered. What caused the obstinate resistance in the windpipe I never could learn.

CASE 10: Little girl age 6. Condyloma in ant. commissure of the larynx, the slightest exposure causing spells of difficult breathing. Intubated, extracted tube on eighth day, pressure of tube having caused some absorption. The symptoms recurring shortly after, tracheotomy was done two years ago. The larynx was then split open and the tumor removed. Now after several years the patient is still well and has a perfect voice.

CASE 11: Woman age 35. Sent to Riverside Hospital for difficult breathing. Found subglottic stenosis, and old laryngeal syphilitic cicatrices. Appointed operation for next day. That night patient had a severe fit of choking. Tracheotomy under cocaine. This was a large fleshy woman, weighing over two hundred pounds, and therefore had a large amount of fat about the neck, which made the operation rather difficult. Tube worn now five years, and will probably wear it the rest of her life. Although she could get along without it there is still slight stenosis, and she fears disaster if she removes it permanently. She wears the tube with comfort, keeping it corked most of the time, nobody being any the wiser by her speech.

CASE 12: Little boy 18 months old, occurring in the practice of Dr. Harris at Tonawanda. Intubated for diphtheria. Tube removed several times during the first eight days, but always to be replaced. The child being poorly nourished, and fearing the formation of granulation tissue, I proposed tracheotomy, which was done. Tube was removed in three weeks and child gradually recovered.

CASE 13: Diphtheria in the practice of Dr. Schladermund. Little child age 15 mos. Brought in from Clarence Center, breathing heavily. Sick several days. No antitoxin had been used because of a faulty diagnosis. Tried to intubate but met with resistance. Several attempts were made with the same result. Tube could not be forced down more than half way. Tracheotomy was done at 11 o'clock at night. Child died at 7 A. M. My assistants were the father and grandmother of the little patient. You can imagine the dread of operating under such circumstances. While feeling for the trachea I ran a retractor hook into my finger which caused some bleeding. Being sensible of the possibility of infection, I felt a peculiar sensation running through me. I tried to counteract the supposed poison with several doses of whiskey that night, which gave me "such a headache" that the infection must have been scared out; at any rate the disease did not develop.

CASE 14: Mr. B, age 60. Was called about 3 o'clock one morning. I found him breathing with difficulty, simulating asthma. Had been having attacks of this kind for several months. This case differed so much from the ordinary attacks of asthma, that I concluded there was some underlying cause, and therefore did not agree with some of the other consulting physicians. However, the patient needed very little care that night. He was always cheerful and ready to crack a joke in his most distressing moments, very unlike an asthmatic. The patient had a very few days respite and my recommendation to open the windpipe was finally adopted, but not until some twenty-four doctors had examined the case and given their opinions. Operated under cocaine.

CASE 15: Little girl age 6, diphtheria. Intubated by Dr. Jas. E. King, but after several weeks, tracheotomy. During a period of six months the child was intubated 50 to 100 times; larynx split the whole length of the cricoid cartilage several times to remove cicatricial tissue which clouded the lumen of the trachea. Twice I did tracheotomy besides and once when this child was choking Dr. Weed, then interne at Riverside Hospital, did an emergency tracheotomy. (Presence of mind which internes seldom possess). Child finally recovered with a perfect voice.

CASE 16: Boy age 16. Gradually suffocating from lateral pressure of fibrous thyroid gasping for "wind" as he called it. After having opened the windpipe a soft catheter had to be used as no ordinary tube would pass beyond the pressure isthmus. Thyroids removed mostly under cocaine, but at last chloroform. Later, mild cataract of both eyes developed, and within a year epilepsy, which is always controlled by bromides.

CASE 17: Boy aged 10 found in extremis; cause diphtheric membrane which filled nose, throat and larynx. Operation with very little anesthetic. Instead of alkaloidal poisoning he needed oxygen. With sufficient antitoxin he gradually recovered.

CASE 18: Woman aged 63 looked much older, suffering for 6 mo. with laryngeal carcinoma. Larynx removed with preliminary tracheotomy. Died same day from supposed hemorrhage.

CASE 19: Woman aged 51, difficult breathing for a week with 7 or 8 choking spells a night. Tracheotomy without anesthetic; patient walked to bed and requested a glass to have a look at the tube in her neck. Larynx was partially removed a week later. Recurrence of growth on opposite side 4 months later. Too late for removal. *X*-Ray treatment finally brought about complete cure.

CASE 20: Man aged 55, saloon keeper claimed to have drunk 200 glasses of beer a day. Had Brights and consequent edema of pharynx and soft palate. Breathed with difficulty so that tracheotomy became necessary without chloroform. Stopped breathing before trachea was open. Could not be brought back to life probably drowned in beer.

CASE 21: Boy aged 3, patient of McIntyre's, accidently sucked a coffee bean into the trachea; this however caused no difficulty in breathing, but the bean was so large that it could not pass out again but could be heard passing up and down whenever the child coughed, which however was not often. He was taken to the Riverside Hospital and the trachea opened under chloroform. The mucus membrane was irritated and cough produced which forced the bean vein it was then caught and extracted.

CASE 22: Lake captain aged 72. Subglottic thickening coming on gradually probably inflammatory deposit. Trachea opened under cocaine. The man was of a most cheerful disposition, cracking jokes continually until the windpipe was opened. Now, after one and one-half years he is perfectly well, not a symptom of any kind and always employed as caretaker of some boat.

One day after these notes were penned he had a sudden hemorrhage and died. Post-mortem disclosed laryngeal carcinoma involving the entire larynx, foul smelling due to large slough.

CASE 23: George Mayes, age 37, patient and brother-in-law of Dr. F. W. Koehler, was troubled with difficult breathing for a year and was making so much noise while asleep that he disturbed the rest of a next door neighbor. Dr. Emil Mayer of New York saw him Nov. 30, 1904, and though there was inflammatory adhesions between the vocal cords. The left false cord overlapped the right and movement was restricted to perhaps 1-16" so that the rest of the larynx could not be examined. Tracheotomy at General Hospital under cocaine. A few weeks later several attempts were made to separate adhesions but without avail, and dilatation was impossible. September 16, 1905, he was again taken to the hospital and with the assistance of Dr. Mayer the larynx was opened, separating the thyroid and cricoid cartilages. A solid mass of apparent fibroid tissue almost closed the lumen of the subglottic windpipe including the left vocal cord; this was thoroughly removed under cocaine and the parts united with silver and silkworm gut; and the tracheal tube replaced. The pathological findings were epitheloma. After a week the patient was treated with the *x*-rays (6 weeks, 14 treatments in all) under which he rapidly improved, but is obliged to wear the tube constantly, there still being too little room for breathing. No lymphatic involvement and larynx freely movable, while before treatment it was fixed.

CASE 24: Little girl age 3 1-2 in practice of Dr. Knisley. Patient apparently healthy began with difficult breathing; doctor suspected diphtheria and injected 3000 units antitoxin; no improvement. Twenty-four hours later I was summoned and found the child comatose, finger nails and lips blue, gasping but slightly for breath at frequent intervals; tried to intubate but could'nt get 2 year tube more than half way down. No obstruction in larynx; as moments were precious, tracheotomy was proposed. There was no time for preparation, either of patient, instruments, or hands. As the child did not stop breathing altogether we proceeded carefully to avoid cutting veins, which bulged like pipe stems. After opening the trachea and inserting a male silver catheter, some minutes passed before there were any signs of reaction, then a slight cough, followed by another until gradually breathing became more regular. Half an hour later when I left the house patient had improved very much. Ten days later the child was playing about the room. Tube removed on the twelfth day. Diagnosis: acute subglottic edema.

CASE 25: Miss MacCauley of Canada, aged 33, consulted me September 6th for periodical hemorrhage coming up, a mouthful and often less. I noticed some blood in the furrows of the base of the tongue where the lingual tonsil was quite large and veins prominent. Thinking the blood came from that source the tonsil was removed and healed nicely. Slight hemorrhage occurred, however, at intervals, but of a mild nature. About a month later, preparatory to her going home, I inspected the parts again and found immediately behind the left arytenoid cartilage, external to the larynx of course and apparently emenating from the esophagus, unmistakable evidence of epithelioma of very small size except that the arytenoid was endematous. A little arterial blood was seen on the surface of the growth, which was about the size of ½". Preliminary to removal of that portion of the larynx as well as the upper esophagus I did tracheotomy November 13, 1905. Although it was my 25th patient it was the easiest one of all. All the structures were so thin that less than an eighth of an inch had to be cut through; the loss of blood was imperceptible. Second operation November 22, 1905 by Dr. Eugene Smith at the Homeopathic Hospital. The larynx was extirpated with as much of the esophagus as seemed soft; however the growth extended below the line of discretion and as the case seemed hopeless anyway the trachea was stitched to the skin and a tube left in the esophagus for feeding purposes. Patient recovered without shock and next day temperature only once reached 100°. She did nicely for 2 weeks. The wound granulating up and looked healthy; then Schluck pneumonia set in from which she suffered a week, when she passed away.

CASE 26: Teacher, age 47, unmarried. May, 1895 had left breast removed for carcinoma; recovered completely. October, 1905 Dr. Morrison referred her to me for a large tumor in her throat which nearly filled the pharynx. Thiswas removed with a cold snare and found to be attached to the lateral wall immediately behind the posterior pillar and slightly above lower palatal margin. This was examined and found to be a round-celled sarcoma. A week later the tonsil on opposite side including cervical glands bulging perceptibly, a piece was removed and examined and found to be lymphadenoma. In four weeks, however, the tonsil had grown so much that it threatened to close up the whole pharyngeal vault. Tracheotomy was then done primarily with a view to lateral pharyngotomy to extirpate the tonsil. The adhesions, however, prevented this and the tonsil was removed as much as possible. The tracheal tube was permitted to remain for the patients good after the tumor reformed. Microscopical diagnosis, spindle-celled sarcoma.

1248 MAIN STREET.

